# Supplementing alpha-tocopherol (vitamin E) and vitamin D3 in high fat diet decrease IL-6 production in murine epididymal adipose tissue and 3T3-L1 adipocytes following LPS stimulation

**DOI:** 10.1186/1476-511X-10-37

**Published:** 2011-02-27

**Authors:** Fábio S Lira, José C Rosa, Claudio A Cunha, Eliane B Ribeiro, Claudia Oller do Nascimento, Lila M Oyama, João F Mota

**Affiliations:** 1 Departamento de Fisiologia, Universidade Federal de São Paulo, Brasil

## Abstract

**Background:**

It is well known that high fat diets (HFDs) induce obesity and an increase in proinflammatory adipokines. Interleukin-6 (IL-6) is considered the major inflammatory mediator in obesity. Obesity is associated with a vitamin deficiency, especially of vitamins E and D3. We examined the effects of vitamin D3 and vitamin E supplementation on levels of IL-6 and IL-10 (as a marker of anti-inflammatory cytokines since, a balance between pro- and anti-inflammatory cytokines is maintained) protein expression in adipose tissue of mice provided with an HFD. Additionally, we measured the effects of vitamin E and vitamin D3 treatment on LPS-stimulated 3T3-L1 adipocytes IL-6 and IL-10 secretion.

**Results:**

IL-6 protein levels and the IL-6/IL-10 ratio were decreased in epididymal white adipose tissue in groups receiving vitamins E and D3 supplementation compared to the HFD group. A 24-hour treatment of vitamin D3 and vitamin E significantly reduced the IL-6 levels in the adipocytes culture medium without affecting IL-10 levels.

**Conclusions:**

Vitamin D3 and vitamin E supplementation in an HFD had an anti-inflammatory effect by decreasing IL-6 production in epididymal adipose tissue in mice and in 3T3-L1 adipocytes stimulated with LPS. Our results suggest that vitamin E and D3 supplementation can be used as an adjunctive therapy to reduce the proinflammatory cytokines present in obese patients.

## Introduction

The incidence of obesity has dramatically increased during the past several decades [1]. In 2006, the World Health Organization estimated that 1.6 billion adults worldwide were overweight and 400 million adults were obese [2]. Although morbidly obese people have a greater food intake than non-obese people, they may suffer from nutritional deficiencies.

High fat diets (HFDs) contribute to the accumulation of adipose tissue, resulting in obesity and possibly insulin resistance. Adipose tissue secretes soluble factors called adipokines, which include pro-inflammatory cytokines. It is known that long-term low systemic inflammation that occurs in obese individuals contributes to the development of metabolic syndrome [3]. Lira et al. [4] reported that body fat correlates with cytokine levels, and it was demonstrated that increased body fat was associated with elevated cytokine levels in obese adolescents. Tumor necrosis factor-α (TNF-α) and interleukin-6 (IL-6), which are increased in obese subjects, may downregulate adiponectin production. In contrast, adiponectin reduces the production and activity of TNF-α and IL-6. IL-6 is considered the major inflammatory mediator in obesity. IL-6 has been implicated in the release of triglycerides and free fatty acids, downregulated lipoprotein lipase, insulin resistance, increased production of reactive oxygen species and decreased nitric oxide generation [5].

IL-10, which is an anti-inflammatory cytokine, may antagonize the actions of IL-6 [5]. Recently, Hong et al. (2010) demonstrated that IL-10 increases insulin sensitivity, protects from obesity-associated macrophage infiltration, prevents increases in proinflammatory cytokines and may prevent the deleterious actions of pro-inflammatory cytokines on insulin signaling and glucose metabolism [6].

Obesity is associated with vitamin deficiencies. Low concentrations of 25-hydroxyvitamin D3 have been associated with an increased risk of diabetes and other cardiovascular disease risk factors [7]. In patients with metabolic syndrome, six weeks of alpha-tocopherol supplementation reduced serum levels of TNF-α [8].

Antioxidant micronutrients have been proposed as adjunctive therapy in patients with diabetes [9]. Indeed, some minerals and vitamins are able to participate indirectly in the reduction of oxidative stress in diabetic patients by improving glycemic control and/or exerting antioxidant activity. For example, Foss [10] speculated that vitamin D deficiency is the cause of obesity, which can be reversed by improving the levels of vitamin D. On the contrary, Czernichow and colleagues [11] demonstrated that the incidence of metabolic syndrome was reduced in healthy individuals that were given an anti-oxidant formula containing vitamin E, vitamin C, beta-carotene, zinc and selenium.

Our hypothesis is that the well established proinflammatory effect of HFD ingestion on white adipose tissue could be reduced by supplementation of vitamin D3 and alpha-tocopherol (vitamin E). The aim of this study was to examine the effects of vitamins D3 and E supplementation on the levels of IL-6 and IL-10 protein expression in adipose tissue of mice provided with a HFD. Additionally, we evaluated the effects of vitamin E and vitamin D3 on 3T3-L1 adipocytes stimulated with LPS in terms of IL-6 and IL-10 secretion.

## Materials and methods

The Experimental Research Committee of the São Paulo Federal University approved all procedures for the care of the animals used in this study (protocol n°2007/0388). Thirty-day-old male Swiss mice were used in this study and were kept under controlled conditions (12 h light: 12 h dark cycle with lights on at 07:00; 22°C ± 1°C). During the experimental period, the animals were group-housed and provided water and a specific diet *ad libitum*. The animals were treated for eight weeks as follows: 1) HFD - Twelve animals were treated for eight weeks with a hyperlipidic diet based on the AIN93 formulation with 20% of the fat from soya beans; 2) HFD Vitamin D3 - Twelve animals were treated for four weeks with a hyperlipidic diet based on the AIN93 formulation with 20% of the fat from soya beans and an additional four weeks with the same diet supplemented with 1,25(OH)_2 _vitamin D3 (0.05 mg/Kg of diet); and 3) HFD Vitamin E - Twelve animals were treated for four weeks with a hyperlipidic diet based on the AIN93 formulation with 20% of the fat from soya beans and an additional four weeks with the same diet supplemented with alpha-tocopherol (0.9 g/kg of diet). The animals' weight and food intake were evaluated weekly.

### Sample collection

After eight weeks the animals were euthanized by decapitation without sedation. Retroperitoneal, mesenteric and epididymal fat pads were removed and weighed to estimate the degree of obesity. Samples of epididymal white adipose tissue were dissected, immediately frozen in liquid nitrogen and stored at -80°C.

### Cell culture

3T3-L1 cells were obtained from the American Type Culture Collection and cultured at a 37°C, 5% CO2 and 95% humidity. The cells were maintained in Dulbecco's Eagle modified medium (DMEM) supplemented with 25 mM glucose, 1.0 mM pyruvate, 4.02 mM L-alanyl-glutamine and 10% fetal bovine serum (FBS) (Gibco). The cells were differentiated 24 h after reaching confluency for 4 days in a medium containing 0.25 μM dexamethasone, 0.5 mM 3-isobutyl-1-methylxanthine, and 5 μg/mL insulin (Sigma). After the cells were differentiated, the cells were cultured for 8 days in growth medium containing 5 μg/ml of insulin.

### Treatment

Eight days after the differentiation, the cells were pretreated with medium containing vitamin D3 (10^-7 ^M) (1,25(OH)_2 _vitamin D3) and vitamin E (50 μM - alpha-tocopherol) for 24 h. Cells were then treated with the same medium along with LPS (75 ng/ml) for an additional 24 h before being harvested. No vitamins were added to the control plates, but the medium was changed. The cultured medium was collected in tubes and stored at -80°C.

### Analysis of IL-6 and IL-10 levels

Adipose tissue samples were carefully rinsed in ice-cold 0.9% NaCl to remove any blood contaminants, snap frozen in liquid nitrogen, and stored at -80°C. Frozen tissue (0.1 g - 0.3 g) was homogenized in RIPA buffer (0.625% Nonidet P-40, 0.625% sodium deoxycholate, 6.25 mM sodium phosphate and 1 mM ethylenediamine tetraacetic acid at pH 7.4) containing 10 mg/ml protease inhibitor cocktail (Sigma-Aldrich, St. Louis, Missouri). Homogenates were centrifuged at 12,000 g for 10 min at 4°C. The supernatant was saved and protein concentration was determined by a Bradford assay (Bio-Rad, Hercules, CA) using bovine serum albumin (BSA) as a standard. Levels of IL-6 and IL-10 in adipose tissue and medium culture were quantified by ELISA (DuoSet ELISA, R & D Systems, Minneapolis, MN). The IL-6 (DY506) and IL-10 (DY522) assay sensitivities were found to be 5.0 pg/ml in the range of 31.2 - 2000 pg/ml. The intra- and inter-assay variability of the IL-6 and IL-10 kits was 2.7-5.2% and 4.9-9.5%, respectively. The intra-assay variability of the IL-6 and IL-10 kits was 2.0-4.2%, and the inter-assay variability was 3.3-6.4%. All samples were run as duplicates, and the mean values are reported.

### Statistical analysis

The results are expressed as a mean ± S.D. For statistical analysis, a one-way ANOVA was used to compare the treatment effects (presence or absence of vitamin D3 and vitamin E). Tukey's test was applied for comparison of the results within the means, and p < 0.05 was considered statistically significant.

## Results

### *In vivo *study

Vitamin D3 and vitamin E supplementation decreased the body weight gain in mice provided with an HFD (39%, p < 0.01 and 48%, p < 0.001, respectively). However, the adiposity index and energy intake were similar among the studied groups (Table 1).

**Table 1:** Body weight again, epididymal adipose tissue weight, adiposity index and cytokines levels

	High Fat	High Fat plus Vit.D	High Fat plus Vit.E
**Body weight gain (g)**	19.95 ± 4.47a	12.08 ± 4.59b	10.40 ± 2.46b
**Energy intake (KJ/100 g b.w)**	10272 ± 584a	10686 ± 495a	10686 ± 495a
**Vitamin E intake (mg)^†^**	16.6 ± 2.4a	18.34 ± 1.17a	202.07 ± 17.88b
**Vitamin D3 intake (mg)^†^**	0.049 ± 0.007a	0.064 ± 0.004b	0.051 ± 0.001a
**Epididymal WAT weight (g)**	1.01 ± 0.21a	0.72 ± 0.18a	0.88 ± 0.34a
**Adiposity index (g)**	1.86 ± 0.50a	1.29 ± 0.35a	1.33 ± 0.55a
**IL-6 (pg/ug protein^-1^)**	115.88 ± 48.01a	66.29 ± 20.97b	44.94 ± 8.49b
**IL-10 (pg/ug protein^-1^)**	28.46 ± 10.18a	43.42 ± 14.06a	32.30 ± 2.24a
**IL-6/IL-10**	3.23 ± 1.55a	1.63 ± 0.68b	1.39 ± 0.27b

Results are expressed as mean value ± SD. **^†^**Vitamin consumption during the study. Different letters indicate statistically significant differences (p < 0.05).

IL-6 protein content in epididymal adipose tissue (WAT) was reduced (p < 0.05) by vitamin D3 (32%) and vitamin E (61%) supplementation in mice treated with HFD. Similar IL-10 WAT protein content was observed among the groups, although the IL-6/IL-10 ratio was reduced in groups supplemented with vitamins in relation to the HFD group, (vitamin D3: 50%, and vitamin E: 57%, p < 0.01) (Table 1).

### *In vitro *study

Twenty-four hours of LPS treatment caused an increase in both IL-6 (approximately 14-fold) and IL-10 protein concentration in 3T3-L1 adipocytes culture medium. The IL-10 protein was not detectable in the control medium.

Twenty-four hours of supplementation with vitamin D3 and vitamin E in the adipocytes stimulated with LPS significantly reduced the levels of IL-6 (88% and 60%, respectively; p < 0.01) in the culture medium without any effect on the IL-10 levels (Table 2). Vitamin D3 and E reduced the IL-6/IL-10 ratio in adipocytes stimulated with LPS (Table 2).

**Table 2:** Cytokine levels in 3T3-L1

	Control	LPS	LPS plus Vit.D	LPS plus Vit.E
**IL-6 (pg/ug protein^-1^)**	0.272 ± 0.034a	36.80 ± 16.08b	4.36 ± 2.16a	14.54 ± 4.95c
**IL-10 (pg/ug protein^-1^)**	ND	55.88 ± 4.84b	56.95 ± 5.25b	58.94 ± 5.50b
**IL-6/IL-10**	-	0.67 ± 0.09a	0.08 ± 0.01b	0.25 ± 0.03b

Results are expressed as mean value ± SD. ND: no detectable. Different letters indicate statistically significant differences (p < 0.05).

## Discussion

Supplementation of vitamin D3 and vitamin E reduced proinflammatory cytokine production by the WAT from the HFD-fed mice. In the 3T3-L1 adipocytes, LPS stimulated IL-6 and IL-10 production. Addition of vitamins D3 and E in the culture medium with LPS significantly reduced IL-6 levels without altering IL-10 levels. These results suggest the importance of vitamins, both *in vivo *and *in vitro*, in controlling the inflammatory state in adipocytes.

Obesity occurs due to a complex combination of multiple environmental and genetic factors. Consumption of highly energetic, fatty, and palatable foods is the most important epidemiological predisposing factors to obesity development [12,13]. Studies have shown that the increase in the production of adipokines, such as PAI-1 [14], TNF-α [15] and IL-6, [16], and the reduction of adiponectin [17] are critically involved in the pathogenesis of obesity.

HFD causes lipotoxic impairment of insulin action and the regulatory mechanisms of body weight [18]. Mice fed with HFD supplemented with vitamin E and vitamin D3 had a slight gain in body weight without changes in the adiposity index and energy intake, which suggested an increase in thermogenesis and metabolic rate.

Several studies have shown an inverse association of serum vitamin D3 levels with adiposity and body mass index [19-22]. In addition, it has been demonstrated that blockade of 3T3-L1 cell differentiation by vitamin D3 involves C/EBPβ suppression, PPARγ upregulation, and antagonist function of PPARγ activity [23,24].

However, Wong et al. [22] demonstrated that vitamin D receptor-null mice have a higher rate of fatty acid beta-oxidation and an up regulation of uncoupling protein (UCP) 1, UCP2 and UCP3 expression in the WAT. They also demonstrated that 1,25-dihydroxyvitamin D3 directly suppressed the expression of the UCPs in primary brown fat culture. They concluded that vitamin D3 is involved in energy metabolism and adipocyte biology *in vivo *in part through regulation of beta-oxidation and UCP expression. The difference between this study and the present study may be due to differences in the protocols that were used.

Furthermore, Sun and Zemel [23] showed that calcitriol increased production of reactive oxygen species (ROS) by inhibiting UCP2 expression and stimulating intracellular calcium influx, resulting in fatty acid synthesis and lipolysis inhibition. However, Zembel and Sun [24] suggested that calcitriol exerts a dose-dependent impact on adipocyte apoptosis, indicating that a low physiological dose inhibits apoptosis and a pharmacological dose stimulates apoptosis. More studies are necessary to understand the effects of HFD with vitamin D3 and vitamin E supplementation on body weight gain and thermogenesis.

The effect of vitamin E supplementation on body mass index is not conclusive. Botella-Carretero and colleagues [25] demonstrated an inverse correlation between vitamin E and body mass index, whereas other studies have reported no correlation [26,27].

Obesity and insulin resistance are associated with low grade chronic systemic inflammation [28]. In models of diet-induced obesity and genetic obesity, the adipose tissue has increased expression of proinflammatory cytokines, such as TNF-α, IL-1, and IL-6 [15,29]. Furthermore, fatty acids increased IL-6 serum concentration and gene expression in adipocytes [30], and a saturated fat-rich diet increased IL-6 concentrations in human adipocytes [31]. The main result of our study is that treatment with vitamin D3 and vitamin E reduces IL-6 levels in epididymal adipose tissue (*in vivo*) and 3T3-L1 adipocytes (*in vitro*).

Low concentrations of vitamin D is the most common vitamin deficiency associated with obesity and may be closely linked to the increase in ROS which activates the inflammatory pathway [7]. There is evidence that insulin resistance results from the proinflammatory cytokines actions released from the adipose tissue, such as TNF-α and IL-6 [32].

Production of IL-6 increases with adiposity, and circulating IL-6 concentrations are highly correlated with percentage of body fat and insulin resistance [33]. Vitamin E supplementation in patients with diabetes decreases the levels of proinflammatory cytokines, such as IL-1, TNF-α, IL-6, and reactive C protein, in serum and stimulated monocytes [34,35]. In addition, high intake of foods rich in vitamin E demonstrated an inverse correlation with levels of C reactive protein and IL-6 [36]. Elevated TNF-α concentrations cause stress oxidation in monocytes which, conversely, increases low density lipoprotein (LDL) oxidation [37]. Oxidative modification of lipoproteins is an essential component of the atherogenic process [34]. Manning et al. (2004) showed that vitamin E supplementation decreased plasma peroxide concentration in obese individuals [26]. In the present study, we demonstrated that a vitamin D3- and vitamin E-supplemented diet decreases IL-6 levels in adipose tissue and in 3T3-L1 adipocytes. Taken together, these results demonstrate that vitamin E and vitamin D3 could have a direct effect on adipocytes.

In conclusion, vitamin D3 and vitamin E supplementation in HFD had an anti-inflammatory effect by decreasing IL-6 production in epididymal adipose tissue in mice and in 3T3-L1 adipocytes stimulated with LPS. Potentially, our results suggest that vitamin E and D3 supplementation can be used as an adjunctive therapy to reduce proinflammatory cytokines levels in obese patients.

## Competing interests

The authors declare that they have no competing interests.

## Authors' contributions

FSL carried out ELISA experiments, performed the statistical analysis and drafted the manuscript. JCR helped to carried out the experiments. CAC helped to carried out the experiments. EBR revised and helped to draft the manuscript. CON conceived of the study, participated in its design and helped to draft the manuscript. LMO conceived of the study, participated in its design, coordination and helped to draft the manuscript. JFM designed the study, carried out the experiments, performed the statistical analysis and drafted the manuscript.

All authors read and approved the final manuscript.
